# Errata

**DOI:** 10.36660/abc.20210556

**Published:** 2021-07-15

**Edição de Março de 2021, vol. 116 (3), págs. 383-392**

No Artigo Original “Valor Prognóstico da Ultrassonografia Pulmonar para Resultados Clínicos em Pacientes com Insuficiência Cardíaca: Uma Revisão Sistemática e Metanálise”, com número de DOI: https://doi.org/10.36660/abc.20190662, publicado no periódico Arquivos Brasileiros de Cardiologia, 116(3):383-392, na página 383, corrigir o nome da instituição Chengdu City First People’s Hospital para: Department of Cardiology, Chengdu First People’s Hospital; da instituição Chengdu Sixth People’s Hospital para: Department of Cardiology, The Sixth People’s Hospital of Chengdu. E corrigir a afiliação do Dr. Min Ma para: Department of Cardiology, The Sixth People’s Hospital of Chengdu.

**Edição de Abril de 2021, vol. 116 (4), págs. 806-811**

No Artigo Original “Aumento da Rigidez Arterial Pulmonar e Comprometimento do Acoplamento Ventrículo Direito-Artéria Pulmonar na SOP”, com número de DOI: https://doi.org/10.36660/abc.20190762, publicado no periódico Arquivos Brasileiros de Cardiologia, 116(4):806-811, na página 806, corrigir o nome do autor Hilmi Sumbul para: Hilmi Erdem Sumbul.

**Edição de Abril de 2021, vol. 116 (4), págs. 844-849**

Na Comunicação Breve “Pacientes Naïve Infectados por HIV Apresentam Disfunção Concomitante com Diminuição de Anticorpos Naturais contra Autoantígenos Derivados da Apolipoproteína B Definidos”, com número de DOI: https://doi.org/10.36660/abc.20200062, publicado no periódico Arquivos Brasileiros de Cardiologia, 116(4):844-849, na página 844, corrigir o título do artigo em português “Pacientes Naïve Infectados por HIV Apresentam Disfunção Concomitante com Diminuição de Anticorpos Naturais contra Autoantígenos Derivados da Apolipoproteína B Definidos” para: “Os Pacientes Naïve Infectados pelo HIV Apresentam DisfunçãoEndotelialConcomitante com a Diminuição de Anticorpos Naturais contra Definidos Autoantígenos Derivados da Apolipoproteína B”.

