# Supplementary material for: Errata
Source: Arq Bras Cardiol. 2021 Jul 15;117(1):265. [Article in Portuguese] doi: 10.36660/abc.20210556 (PMC8294734; doi:10.36660/abc.20210556)
Supplement: Translation

# Translation: Erratum

**March 2021 Issue, vol. 116 (3), pages 383-392**

In Original Article “Prognostic Value of Lung Ultrasound for Clinical Outcomes in Heart Failure Patients: A Systematic Review and Meta-Analysis”, with DOI number: https://doi.org/10.36660/abc.20190662, published in the Journal Arquivos Brasileiros de Cardiologia, 116(3):383-392, on page 383, correct the name of the institution Chengdu City First People’s Hospital to: Department of Cardiology, Chengdu First People’s Hospital; correct the name of the institution Chengdu Sixth People’s Hospital to: Department of Cardiology, The Sixth People’s Hospital of Chengdu. And correct Dr. Min Ma’s affiliation to: Department of Cardiology, The Sixth People’s Hospital of Chengdu.

**April 2021 Issue, vol. 116 (4), págs. 806-811**

In Original Article “Increased Pulmonary Arterial Stiffness and Impaired Right Ventricle-Pulmonary Artery Coupling In PCOS”, with DOI number: https://doi.org/10.36660/abc.20190762, published in the Journal Arquivos Brasileiros de Cardiologia, 116(4):806-811, on page 806, corrigir o nome do autor Hilmi Sumbul para: Hilmi Erdem Sumbul.

**April 2021 Issue, vol. 116 (4), págs. 844-849**

In Brief Communication “Pacientes Naïve Infectados por HIV Apresentam Disfunção Concomitante com Diminuição de Anticorpos Naturais contra Autoantígenos Derivados da Apolipoproteína B Definidos”, with DOI number: https://doi.org/10.36660/abc.20200062, published in the Journal Arquivos Brasileiros de Cardiologia, on page 844, correct the title of the article in Portuguese “Pacientes Naïve Infectados por HIV Apresentam Disfunção Concomitante com Diminuição de Anticorpos Naturais contra Autoantígenos Derivados da Apolipoproteína B Definidos” to: “Os Pacientes Naïve Infectados pelo HIV Apresentam Disfunção Endotelial Concomitante com a Diminuição de Anticorpos Naturais contra Definidos Autoantígenos Derivados da Apolipoproteína B”.
